# *Streptococcus tigurinus* is frequent among *gtfR*-negative *Streptococcus oralis* isolates and in the human oral cavity, but highly virulent strains are uncommon

**DOI:** 10.1080/20002297.2017.1307079

**Published:** 2017-04-20

**Authors:** Georg Conrads, Svenja Barth, Maureen Möckel, Lucas Lenz, Mark van der Linden, Karsten Henne

**Affiliations:** ^a^ Division of Oral Microbiology and Immunology, Department of Operative Dentistry, Periodontology and Preventive Dentistry, RWTH Aachen University Hospital, Aachen, Germany; ^b^ Institute of Medical Microbiology and National Reference Center for Streptococci, RWTH Aachen University Hospital, Aachen, Germany

**Keywords:** Mitis group streptococci, endocarditis, housekeeping genes, 16S rRNA gene, phylogeny, glycosyltransferase, pathogenomic profiling, multidimensional scaling

## Abstract

*Streptococcus tigurinus* is a new member of the Mitis group and is associated with infective endocarditis. Low and high virulent variants have been described. A search was made in the national reference collection of endocarditis isolates for *S. tigurinus*–like strains by sequencing housekeeping genes (16S rRNA-gene, *gdh, groEL, sodA*). The strains were further profiled by polymerase chain reaction (PCR) targeting a choice of virulence genes (*rib*-like, *cshA*-like, *gtfR, int, pitA, hylA*). To study the prevalence and abundance of *S. tigurinus* in the saliva and on the mucosal membranes of 35 healthy adults, PCRs detecting two variants of the 16S operon and virulence genes were applied. Among the endocarditis isolates, eight strains (all *gtfR*-negative and former *S. oralis*) holding the specific *S. tigurinus* 16S motif were found, but the pattern of genes related to high virulence found in the *S. tigurinus* type strain could not be detected in any of these strains. A close phylogenetic proximity between *S. tigurinus* and *S. oralis* was observed, with intersectional hybrid strains formed. This was supported by concatenated housekeeping sequences, *in silico* DNA–DNA hybridization, pathogenomic profiling, and multidimensional scaling. In the oral samples, *S. tigurinus* could be detected frequently, especially in the most common operon variant, but none of the type strain–related virulence factors were found. Low virulent *S. tigurinus*–like strains can be found frequently and in high prevalence (66%) and abundance (12.5%) in the oral cavity of healthy adults. In strain collections, they are among the formerly known *gtfR*-negative *S. oralis*. Highly virulent strains seem to be uncommon. Though closely related, *S. oralis* and *S. tigurinus* can be separated by the presence or absence of *gtfR* and dextran production. Hybrids of both species can be found. The variable arsenal of virulence genes found in this study emphasizes the genetic plasticity of Mitis group streptococci.

## Introduction

The precise description and classification of prokaryotic microorganisms has never been an easy task [1]. One reason for the difficulties might be that the zoological definition of a species as ‘groups of interbreeding or potentially interbreeding natural populations that are reproductively isolated from other such groups’ cannot be applied to prokaryotes [2]. Nevertheless, a kind of (man-made) rule of thumb has been established, stating that strains of *Bacteria* or *Archaea* possessing 16S rRNA-gene sequences with >97% identity belong to the same species, but this needs to be checked by DNA–DNA hybridization. This concept has been updated, stating that the cutoff of 97% was too low and can be raised to 98.7% [3]. However, a proof by DNA–DNA hybridization, where a rate >70% is accepted to be indicative for the same species, is still useful. One problem here is that – especially in some taxa such as oral fusobacteria [4] or viridans streptococci [5] – the exact border between species is not well defined (or definable), and this interferes with the sometimes compulsive intention of scientists to describe a species precisely.

Within the genus *Streptococcus*, the classification of members of the Mitis group is especially challenging, as it shows extensive sequence polymorphisms resulting in intra-species DNA–DNA hybridization rates <70% [6,7]. *Streptococcus tigurinus* is a recently discovered oral pathogen, which is able to cause infective endocarditis, meningitis, and spondylodiscitis [8–10]. After the first isolates (AZ_3a [type strain, synonym DSM 24864], AZ_1-AZ_15) were described in Zürich, Switzerland, in 2012 [10], additional *S. tigurinus* isolates were found again in Switzerland (strain 1366 with small colony variants nos. 2425 and 2426), in Japan (two isolates of infective endocarditis [11]), in India (single isolate from the oral cavity of a periodontitis patient [12]), again in Switzerland (several isolates from the oral cavity [13], again in Japan (culture-negative endocarditis case [8] and one from bacteremia [14]), in Spain (endocarditis case [15]), in France (two endocarditis cases [16]), and in Washington, USA, where 14 isolates were recovered from numerous body sides, expanding the spectrum of associated diseases [17]. Up to November 2016, seven *S. tigurinus* genome sequences (strains AZ_3a, 1366, 2425, 2426, DGIIBVI and JPIBVI, UC5873) have been made accessible in GenBank [18–20].

Among the Mitis group reference strains, *Streptococcus oralis* ATCC 35037^T^ and ATCC 49296, ATCC 6249 (formerly *Streptococcus mitis*), and *Streptococcus dentisani* DSM 27088^T^ are the nearest relatives to *S. tigurinus*, while *S*. *mitis* strains, including ATCC 49456^T^, as well as *Streptococcus infantis* ATCC 700779^T^ and *Streptococcus peroris* ATCC 700780^T^, are more distantly related [21]. *S. mitis* ATCC 15914 was reclassified as *S. tigurinus* recently. Confusingly, an ongoing NCBI genome sequencing BioProject (see https://gold.jgi.doe.gov/project?id=33720) on this strain is named ‘*S. sanguinis* ATCC 15914’.

The strain collection of the National Reference Center for Streptococci in Aachen (Germany) contained 18 previously identified *S. oralis*, four *S. infantis*, and one ‘S. tigurinus’ isolates (pre-identified by *sodA*-sequencing) from proven cases of infective endocarditis. The aims of this study were first to screen these strains for the newly described *S. tigurinus* by comparing four different housekeeping genes and, second, to profile a choice of their virulence genes. Furthermore, by applying *S. tigurinus–* and virulence factor–specific polymerase chain reactions (PCRs), the prevalence, abundance, and virulence of this species in saliva and on buccal mucosal membranes of 35 healthy adults were determined.

## Materials and methods

### Identification of endocarditis-associated strains and pathogenomic profiling

#### Strains and phylogenetic analysis

DNA was extracted from *S. oralis* SN 16495, SN 17127, SN 28194, SN 31376, SN 37569, SN 37737, SN 39325, SN 40525, SN 45448, SN 48861, SN 50746, SN 51446, SN 54788, SN 58364, SN 58746, SN 59433, SN 60579, SN 63707, *S. infantis* SN 54787, SN 57625, SN 17128, SN 19640, ‘S. tigurinus’ SN 62386 (all proven and epidemiologically unrelated endocarditis isolates collected by the National Reference Center for Streptococci, Aachen), and from four *S. tigurinus* reference strains, including type strain AZ_3a, as well as small colony variants (2425, 2426) of parental strain 1366 [19] kindly provided by A. Zbinden (Zürich). The 16S rRNA-gene (*Escherichia coli* position 27–557) and three other housekeeping genes known for their species-specific resolution [22,23], *gdh* (coding for glucose-6-phosphate 1-dehydrogenase, gene position 745–1404), *groEL* (coding for a heat shock protein, molecular chaperone GroEL, gene position 555–1312), and *sodA* (coding for a superoxide dismutase, gene position 36–471) were amplified and sequenced. The ambiguity-free and most informative core-fragment of each gene (a length of 442, 541, 678, and 398 bp, respectively, summing up to 2,059 bp in total) was analyzed. In the case of the 16S rRNA-gene of Mitis group streptococci, complete sequencing does not lead to a concentration but to a dilution of information, and thus sequencing of the V1–V2 region (covering all annealing sites of specific oligonucleotides described so far [13]) was preferred as most efficient. Sequences were compared *in silico* with each other and with reference strain genomes of related species available in GenBank. In addition, a concatemeric tree (‘housekeeping tree’ based on all 2,059 bp, maximum likelihood) of the genes mentioned above was built to estimate phylogenetic relatedness with reliable bootstrap values.

#### In silico DNA–DNA hybridization

To investigate further the phylogenetic relationship among *S. tigurinus*, *S. oralis*, *S. infantis*, *S. peroris*, and *S. mitis*, *in silico* DNA–DNA hybridization was performed using the genome-to-genome distance calculator (GGDC2) provided on the DSMZ website [24]. This tool is able to calculate both the genomic distance and the probability that two strains, compared on genome level, belong to the same species defined by a DNA–DNA hybridization rate of >70%.

#### Multidimensional scaling of distance data

To visualize the actual phylogenetic distance between species based on *in silico* DNA–DNA hybridization (GGDC2) and concatemer data (16S/*gdh*/*groEL*/*sodA*, maximum composite likelihood), multidimensional scaling (MDS) was performed using the software ‘orange data mining’ (version 3.2, and explained by Demsar et al. [25]). In brief, a principal component analysis (Torgerson) was used to initialize the plot and – in (maximal) 10^6^ iterations – the two-dimensional distance was iteratively optimized.

#### In silico *pathogenomic profiling*

To investigate virulence gene profiles further across multiple strains of the Mitis group, including *S. tigurinus*, *S. oralis*, *S. infantis*, and *S. peroris*, the Pathogenomic Profiling Tool (PathoProT) of StreptoBase was used (http://streptococcus.um.edu.my and explained by Zheng et al. [20]).

#### In vitro *pathogenomic profiling*

The presence of six virulence-associated genes was investigated using self-designed PCR assays (Table 1). These genes were *rib*-like (*R*esistance to protease, protective *I*mmunity, originally described in invasive serotype III group *B*-streptococci (*Streptococcus agalactiae*), coding for an M6- or M28-like repetitive protein [26,27]), *cshA-*like (coding for a fibronectin-binding protein originally described in *Streptococcus gordonii* [28,29]), *pitA* (coding for a metal ion-depending adhesin [MIDAS] with a Willebrand factor type A, pfam13519 or VWA_2 domain also found in second type pili [PI-2] of *Streptococcus pneumoniae* [30,31]), *hyl*A (coding for an enzyme involved in degradation of hyaluronate described in *S. pneumoniae* [31]), and *int* (encoding an integrase of a transposable element found in the genome sequence of the *S. tigurinus* type strain). Furthermore, the *gtfR* gene (coding for a glycosyltransferase type GtfR) was analyzed, as it plays an important role in the adhesion of *S. oralis* but – interestingly enough and unusually for viridans streptococci – is described to be present in only 51% of *S. oralis* strains [22]. All PCR conditions are summarized in Table 1. The actual expression of the *gtfR* gene, resulting in dextran production, was tested on sucrose-enriched (5%) Columbia Blood Agar or Mitis Salivarius Agar.

**Table 1.** Primers and polymerase chain reaction conditions used in this study.

Gene	Primer	Sequence	Annealing temperature/elongation time	Literature
16S, *rrnA*	pF1	AGAGTTTGATCCTGGCTCAG	55°C/60 sec	[32]
	pR4	CCAGCAGCCGCGGTAATAC		[33]
*sodA*	sodA-F	TRCAYCATGAYAARCACCAT	50°C/60 sec	[34]
	sodA-R	ARRTARTAMGCRTGYTCCCARACRTC		[34]
*groEL*	StreptogroELd	GAHGTNGTIGAAGGIATGCA	52°C/60 sec	[35]
	StreptogroELr	ATTTGRCGIAYWGGYTCTTC		[35]
*gdh*	GDHsoralisF	ATGGACAAACCAGCTAGCTT	55°C/60 sec	[6]
	GDHsoralisR	GCTTGTGGTCCCATGCTTCC		[6]
*gtfR*	gtfRSoralisF	TCCCGGTCAGCAAACTCCAGCC	52°C/60 sec	[36]
	gtfRSoralisR	GCAACCTTTGGATTTGCAAC		[36]
*cshA-*like	CshA-F	AAGTACAAGGTGCAGATG	46°C/40 sec	This study
	CshA-R	CATTTGCAGTAGCTATCG		This study
*rib-*like	RibF	GTGACYTACCCAGATGG	47°C/30 sec	This study
	RibR1	CAATWGAATCTTCWGCC		This study
*pitA*-like	pitAF	ATTWSTCCAATTCAGGGGC	55°C/45 sec	This study
	pitAR	AATGCCCGCGTCCATACC		This study
*integrase*	InGraF	CTCATGATGTCAAAGAG	46°C/40 sec	This study
	InGraR	GATTTGGAAYCGTGTGG		This study
*hyl*A-like	hylAF	CAGCAAAGGGATGCCAAG	60°C /60 sec	This study
	hylAR	CCTTCCAGTTCCTGGTTTAAC		This study
Tigurinus *16S*, *rRNA*	TIGF_CTT_	AGAACGCTGAAGCTTGGT	55°C /30 sec	This study
	TIGF_AGA_	GTAGAACGCTGAAGAGAGG		This study
	TIGR	ATTATCATGCGATAATCAATT		This study
Mitis-group *sodA*	sodA-MSF	GCMAAYGCAGCTCTTGAA	58°C /35 sec	This study
	sodA-MSR	ACCAACCAHGCCCATCCA		This study

Other parameters: initial denaturation 2 min, 30 cycles, denaturation 30 sec, annealing and elongation see Table, final elongation 10 min. For the *rib*-like PCR, *Streptococcus agalactiae* serotype III (ATCC10202) and *Streptococcus pyogenes* M28 (ATCC1091) served as additional positive controls. As *rib* is a repetitive gene, several amplicons of different length may occur.

### In situ detection of S. tigurinus variants

To determine the prevalence, abundance, and virulence gene profile of *S. tigurinus* in the oral cavity as its natural habitat known so far, saliva and buccal swab samples of 35 healthy young adults (see study population for details) were screened for the presence of (a) all bacteria, (b) all Mitis group streptococci, (c) *S. tigurinus* in two 16S operon variants (with AGA- and CTT-motif; see below and Supplementary Figure S1), and (d) a selection of six *S. tigurinus*–associated virulence genes.

#### Study population and sampling

As probands, dental students of the University Hospital (Aachen) were recruited. Subjects were included if they had no or minimal signs of gingival inflammation and demineralization. In total, 35 students (23–32 years of age, *M*_age_ = 25.3 years; 11 male) fulfilled these criteria. All participants were free of systemic diseases, and none of them had taken any antibiotics within the last 3 months. Participating subjects were informed about the microbial and molecular biological analysis and signed an informed consent that had been approved in accordance with the guidelines of the Ethics Committee of the University Hospital (Aachen, Germany). A volume of 1.5 mL of freshly stimulated saliva, as well as swab samples of both buccal mucosae, were collected from all participants. Bacterial cells were recovered by vortexing with glass beads (diameter 1–2 mm) and centrifugation. All suspensions were then stored frozen at –72°C until further analysis.

#### DNA extraction and quantitative PCR

After thawing, cell suspensions were washed and recovered. To dissolve the salivary mucus, an incubation step with Bioténe® (PBF Oral Rinse, SKB, containing biofilm matrix digesting enzymes such as mutanase and dextranase) for 30 min at 37°C and additional washing ensued. After addition of 20 µL of a lysozyme/mutanolysin solution (0.3 mg of lysozyme + 10 IU of mutanolysin) to the pellet, a 30 min incubation step at 37°C preceded DNA extraction and purification using the QIAamp DNA Mini kit (‘tissue protocol’; Qiagen, Hilden, Germany) according to the manufacturer’s instructions. One microliter of purified DNA (from standards and from samples) was used as the template for real-time quantitative PCR (qPCR) on the Light Cycler 2.0 system (Roche, Mannheim, Germany). The total number of bacterial genome equivalents per microliter of DNA extract (referred to as ‘bacterial cell counts’) was measured according to Nadkarni et al. [37] using *S. tigurinus* AZ_3a DNA as standard. Accordingly, total cell counts of Mitis group streptococci, *S. tigurinus* holding the AGA-motif in the 16S rRNA gene, and *S. tigurinus* holding the CTT-motif in the 16S rRNA gene were measured using *S. tigurinus* AZ_3a (AGA-version) and *S. tigurinus* 1366 (CTT-version) as standards and applying the primers and protocols listed in Table 1. The identity of amplicons was verified by spot-check sequencing using the Applied Biosystems 310 DNA sequencer (Applied Biosystems, Foster City, CA). All calculations were carried out using Microsoft® Excel 2010.

## Results

### Identification of endocarditis-associated strains and pathogenomic profiling

**Figure 1.**
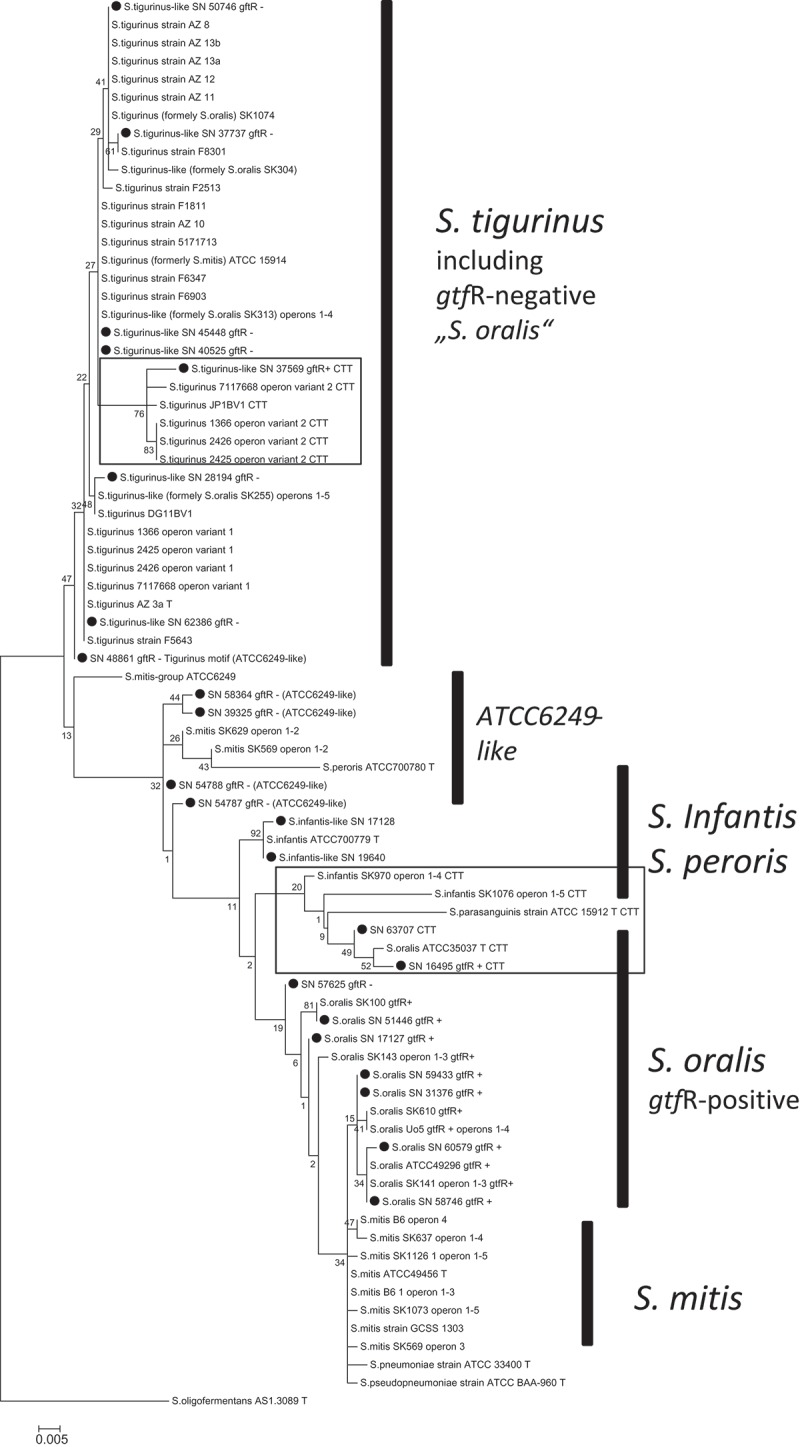
Phylogenetic tree based on the 16S rRNA-gene of 73 Mitis group streptococci. Program MEGA6, maximum likelihood algorithm subsequently to ClustalW-alignment, standard preferences, 100 bootstraps [38]; ●, SN strains from the collection; CTT-motif (strains framed hold a variant of the 16S rRNA gene characterized by a CTT-motif). *Streptococcus oligofermentans* strain AS 1.3089^T^ was used as the outgroup.

Among the endocarditis isolates tested, eight *S. tigurinus*–like strains (SN 28194, SN 37569, SN 37737, SN 40525, SN 45448, SN 48861, SN 50746, and SN 62386) could be easily identified by a specific 16S motif starting at *E. coli* position 176–184. In comparison to all published *S. tigurinus*–like 16S sequences so far, this motif demonstrated some variations, forming the consensus signature AAT(G/T)GATTATCGCATGAT(A/G). Whereas the G/T alteration was frequently found, and may even vary within a strain from operon to operon, the A/G alteration was only found in strain 7117668 (KT780462, French isolate [16]). The 16S rRNA gene-based tree with 73 Mitis group strains (a few represented by two operon variants) and *S. oligofermentans* strain AS 1.3089^T^ as an outgroup (see Figure 1 constructed by MEGA6 [38]) demonstrated a distinct *S. tigurinus*–like cluster, including the eight endocarditis isolates of the collection (labelled ●), 23 *S. tigurinus* strains of other origin, but also four genome-sequenced *gtfR*-negative ‘*S. oralis*’ strains (SK255, SK304, SK313, SK1074; corresponding accession nos. NZ_AFNM00000000.1, NZ_ALJN01000025.1, AFUU00000000.1, NZ_AICT00000000.1). Because of the close phylogenetic relationship with the *S. tigurinus* type strain – according to 16S, housekeeping genes and whole genome (see below) data –these strains were further named ‘*S. tigurinus*–like (formerly *S. oralis*)’.

Interestingly, a few *S. tigurinus* or –like isolates (1366, 2425, 2426, SN 37569 from the collection; strain 7117668 from a French collection) hold two 16S rRNA gene operon variants, one very common AGAGGAGCTTGCTCTTCTT (which is abbreviated to ‘AGA-motif’) and one with the sequence CTTGGTGCTTGCDCCGAGC (which is abbreviated to ‘CTT-motif’) both starting at *E. coli* position 77 (or 69 counting from the 5ʹ end of primer F1). Of even more interest, the same CTT-motif was found in a few strains of closely related species at the same position, including the *S. oralis* type strain ATCC 35037^T^, the *S. parasanguinis* type strain ATCC 15912^T^ (in all 16S rRNA-operons), the *S. infantis* strains SK970 and SK1076 (in all 16S rRNA-operons, whereas *S. infantis* ATCC 700779^T^ holds a 16S rRNA operon variant with AGA-motif), and strains SN 16495 and SN 63707 of the collection. An overview of these results is given in Figure 1 where strains with a 16S CTT-motif are framed. Other clusters were formed by *S. infantis*–*S. peroris*, *S. oralis* (*gtfR*-positive strains only), *S. mitis*, and a few strains together with ATCC 6249, the latter of which were further named ‘ATCC 6249-like’.

**Figure 2.**
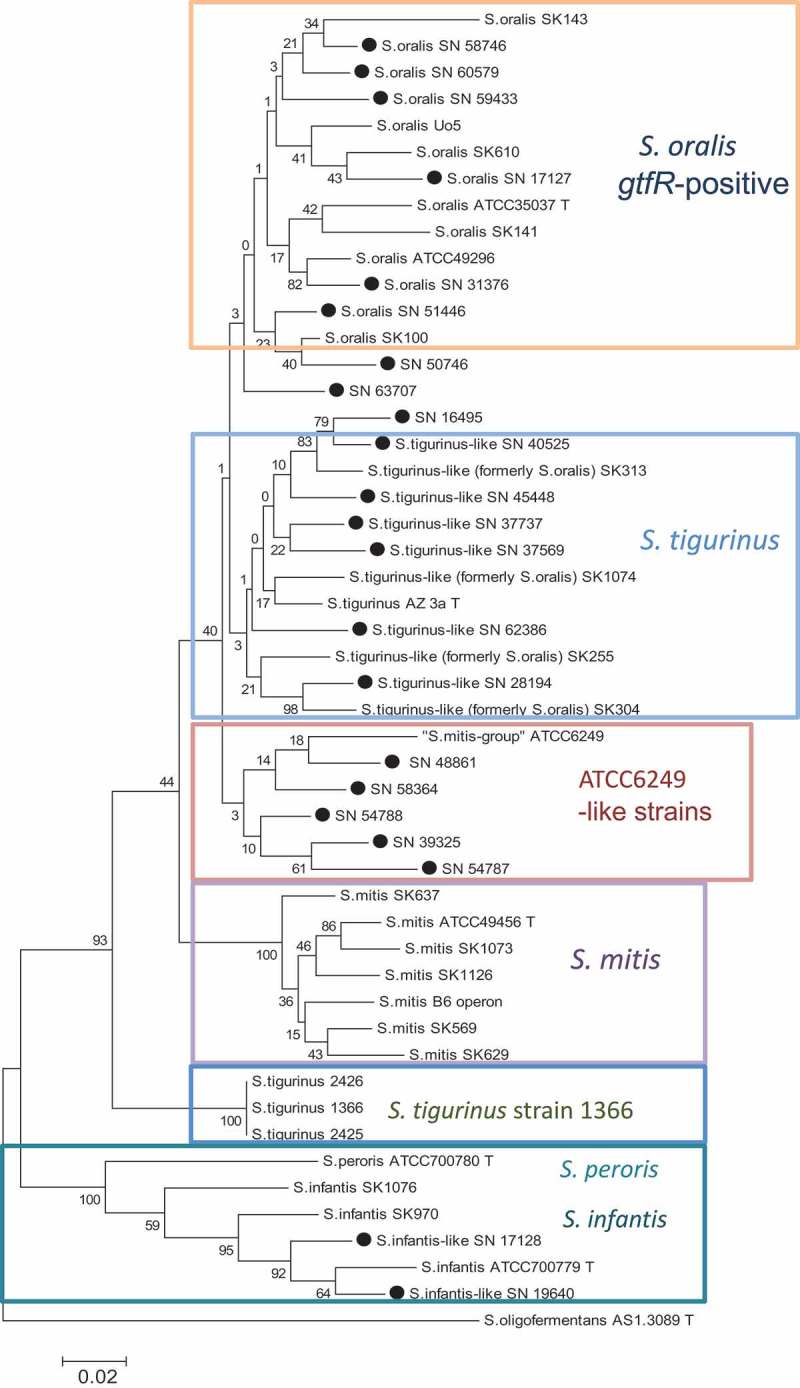
Phylogenetic tree based on the 16S/*gdh*/*groEL*/*sodA*-concatenated sequences of 50 Mitis group streptococci. Program MEGA6, maximum likelihood algorithm subsequently to ClustalW-alignment, standard preferences, 100 bootstraps [38]; ●, SN strains from the collection. *S. oligofermentans* strain AS 1.3089^T^ was used as the outgroup.

To some extent, the Mitis group phylogeny based on the 16S gene was found to be reflected in *gdh-*, *groEL-*, and *sodA*-derived trees (Supplementary Figs. S2–S4). However, whereas *S. infantis* (together with *S. peroris*, a species that is based on a single strain – a singleton) and *S. mitis* form relatively distinct clusters (confirmed by DNA–DNA hybridization; see below and Table 2), *S. tigurinus*–like strains are positioned in close proximity to or even intermingling with *S. oralis*, challenging the species definition. However, a concatemeric tree constructed by combining 16S, *gdh, groEL*, and *sodA* sequence data was able to separate *S. tigurinus* from *S. oralis* further, leaving *S. mitis* and *S. infantis–S. peroris* as distinct clusters (Figure 2). From this concatemeric tree, two other phylogentical characteristics became obvious: (a) linker strains (hybrids) of *S. oralis* and *S. tigurinus* do exist, such as SN 63707; (b) *S. tigurinus* 1366 (and its progenies 2425 and 2426) is relatively distantly related to type strain AZ_3a; (c) ATCC 6249 together with five of the endocarditis isolates (SN 39325, SN 48861, SN 54787, SN 54788, and SN 58364) form another distinct cluster.

**Table 2.** Probability matrix comparing strains within the *Streptococcus tigurinu*s–*Streptococcus orali*s supercluster with each other and with closely related species (*Streptococcus infantis, Streptococcus peroris, Streptococcus miti*s). Numbers show the probability that two strains compared at the genome level *in silico* are of the same species [24]. Boxes indicate probabilities of *gtfR*-negative or *gtfR*-positive *S. tigurinu*s–*S. orali*s strains and the outgrouping of ATCC 6249, respectively.

To investigate further the phylogenetic relationship among *S. tigurinus*, *S. oralis*, *S. infantis*, *S. peroris*, and *S. mitis*, *in silico* DNA–DNA hybridization was performed, calculating a matrix that indicated the probability that both strains belong to the same species [24]. The results are presented in Table 2. For background information, two identical strains usually result in a probability of 98%, and strains of a well-defined species, such as *S. pyogenes*, in a probability between 94 and 98%. In contrast, the *S. oralis* strains showed a reduced probability between 38 and 51% of belonging to the same species. *S. tigurinus*, including –like strains, also showed a reduced probability between 22 and 72%, with the small-colony variant strain 1366 even producing values as low as 21%. Comparing *S. tigurinus*(–like) with *S. oralis* strains resulted in a probability between 14 and 24%. Taken together, the results demonstrate the heterogeneity within the *S. tigurinus*–*S. oralis* supercluster at the genome level, again challenging the species definition.

**Figure 3.**
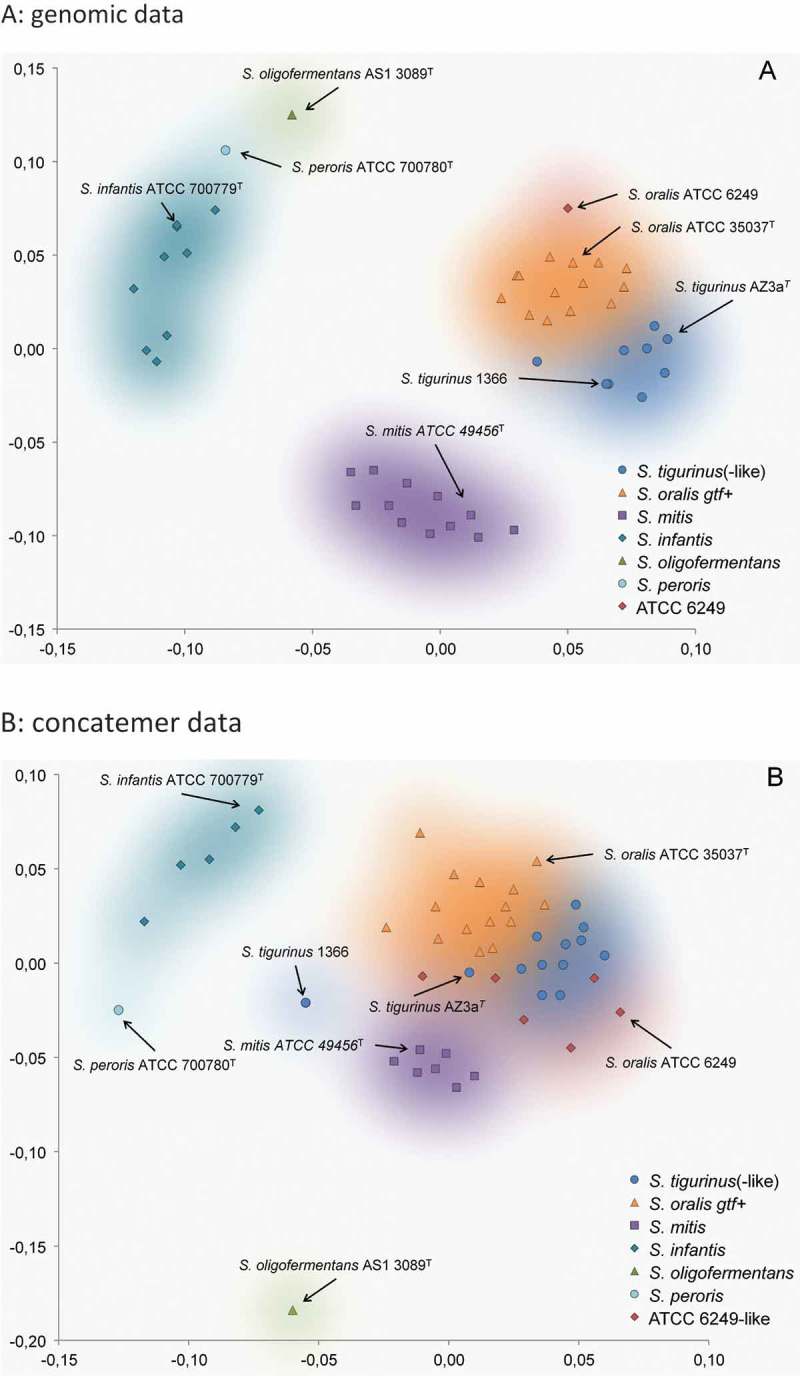
Two-dimensional representation of a multidimensional scaling (MDS, Torgerson scaling). (a) Plot is based on genomic data of selected Mitis group strains. Genomic distances were calculated by *in silico* DNA–DNA hybridization (GGDC2 algorithm [18]). (b) Plot is based on concatemer data (16S/gdh/*groEL*/*sodA*) of selected Mitis group strains, including the SN strains from the collection. Distances were calculated with the maximum composite likelihood algorithm. *S. oligofermentans* strain AS 1.3089^T^ was used as the outgroup in both plots.

The MDS visualization of genomic distances of M-clade streptococcal species revealed clustering (Figure 3(a)). While *S. infantis* (together with *S. peroris*) and *S. mitis* formed distant clusters, *S. oralis* and *S. tigurinus* were found to be closely related. *S. tigurinus* genomes and those genomes classified as *S. tigurinus*–like formed one cluster in direct vicinity to the cluster build by *gtfR*-positive *S. oralis* strains. Reference strain ATCC 6249 – recently reclassified as *S. oralis* – can be found at the edge of the *S. oralis* cluster. In comparison to the genomic distance, the MDS plot of the concatemer data of four in series connected partial housekeeping genes showed that most species distinctions were retained, albeit with some rearrangement of the spatial distributions (Figure 3(b)). *S. infantis* (together with *S. peroris*) formed a cluster at a greater distance to *S. mitis*, *S. oralis*, and *S. tigurinus*, with the latter two converging. Strain *S. tigurinus* 1366 and its derivatives 2425 and 2426 exhibited a great distance, not only to the *S. tigurinus* type strain AZ_3a, but also to all other clusters of the Mitis group. *S. oralis* ATCC 6249 (together with five ATCC6249-like isolates of the collection) is again found at the edge of the *S. oralis–S. tigurinus* supercluster, but this time it is more adjacent to *S. tigurinus*. In both MDS plots, *gtfR*-positive *S. oralis* and *gtfR*-negative *S. tigurinus*(–like) strains formed separate clusters but still in close contact (like the two halves of a coffee bean). An intermingling between both species can be observed and linking (or hybrid) strains identified (compare with above and Table 3).

**Table 3.** Presence of the 16S Tigurinus motif, the 16S CTT-motif, and six virulence-associated genes among endocarditis strains in the collection (SN) and *S. tigurinu*s reference strains.

Strain	Tigurinus-motif	CTT-motif	*rib*-like	*int*	*cshA-*like	*pitA*-like, *hylA*-like^a^	*gtfR*
*S. oralis* ATCC 35037- resp. ATCC 49296-like strains according to 16S and housekeeping genes							
SN 17127	Negative	Negative	Negative	Negative	**Positive**	Negative	**Positive**
SN 31376	Negative	Negative	Negative	Negative	**Positive**	Negative	**Positive**
SN 51446	Negative	Negative	Negative	Negative	**Positive**	Negative	**Positive**
SN 60579	Negative	Negative	Negative	Negative	**Positive**	Negative	**Positive**
SN 58746	Negative	Negative	Negative	Negative	Negative	Negative	**Positive**
SN 59433	Negative	Negative	Negative	Negative	Negative	Negative	**Positive**
*S. oralis*–*S. tigurinus* linker strains							
SN 16495	Negative	**positive**	Negative	Negative	**Positive**	Negative	**Positive**
SN 50746	**Positive**	Negative	Negative	Negative	Negative	**positive**	Negative
SN 63707	Negative	**Positive**	Negative	Negative	**Positive**	Negative	Negative
*S. tigurinus*–like strains according to 16S and housekeeping genes							
SN 37569	**Positive**	**Positive**	Negative	Negative	Negative	Negative	**Positive**
SN 28194	**Positive**	Negative	Negative	Negative	Negative	Negative	Negative
SN 37737	**Positive**	Negative	Negative	Negative	Negative	Negative	Negative
SN 40525	**Positive**	Negative	Negative	Negative	Negative	Negative	Negative
SN 45448	**Positive**	Negative	Negative	Negative	Negative	Negative	Negative
SN 62386	**Positive**	Negative	**Positive**	**Positive**	Negative	Negative	Negative
*S. tigurinus* reference strains							
AZ_3a^T^	**Positive**	Negative	**Positive**	**Positive**	**Positive**	**Positive**	Negative
1366	**Positive**	**Positive**	Negative	Negative	**Positive**	Negative	Negative
2425	**Positive**	**Positive**	Negative	Negative	**Positive**	Negative	Negative
2426	**Positive**	**Positive**	Negative	Negative	**Positive**	Negative	Negative
ATCC 6249-like strains according to 16S and housekeeping genes							
SN 39325	Negative	Negative	Negative	Negative	Negative	Negative	Negative
SN 48861	**Positive**	Negative	Negative	Negative	Negative	Negative	Negative
SN 54787	Negative	Negative	Negative	Negative	Negative	Negative	Negative
SN 54788	Negative	Negative	Negative	Negative	Negative	Negative	Negative
SN 58364	Negative	Negative	Negative	Negative	Negative	Negative	Negative
*S. infantis*–like strains according to 16S and housekeeping genes							
SN 17128	Negative	Negative	Negative	Negative	Negative	Negative	Negative
SN 19640	Negative	Negative	Negative	Negative	Negative	Negative	Negative
SN 57625^b^	Negative	Negative	Negative	Negative	Negative	Negative	Negative
							

All typical *S. oralis* strains hold *gtfR*, and all *S. tigurinus*–like strains do not hold *gft*R. Three strains (SN 16495, SN 50746, and SN 63707) are placed between *S. oralis* and *S. tigurinus* according to housekeeping gene information and might be hybrids. Five strains are related to ATCC 6249 according to housekeeping genes, which could be evidence for another subclade or ‘(sub-)species’.

^a^These genes always appeared together.

^b^In strain SN 57625, the *gdh* could not be amplified.

**Figure 4.**
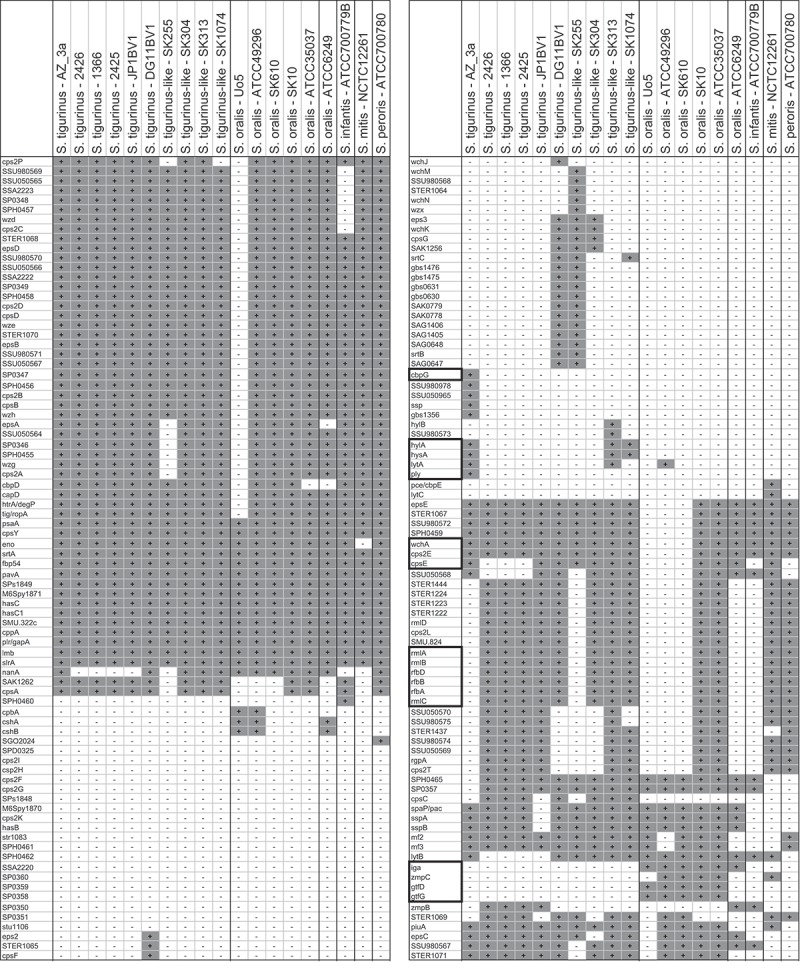
An informative heat map of streptococcal virulence genes generated by the PathoProT tool (StreptoBase). Presence of a virulence gene is labeled in gray and absence in white. Note that ‘absence’ is quite threshold dependent. The standard preferences used here are thresholds for sequence identity and completeness of both >50%. Genes framed in bold are further discussed in the text.

More insights were obtained by applying the recently published Pathogenomic Profiling Tool (PathoProT of StreptoBase [20]) resulting in an informative heat map matrix (Figure 4). This tool screens for the presence of virulence genes in a selection of published streptococcal genomes. In principal, the presence and absence of the virulence gene is labeled differentially, but this is threshold dependent (both of sequence identity and completeness) as the BLAST algorithm is used. The results underline the uniqueness of the *S. tigurinus* type strain AZ_3a. This strain possesses a combination of virulence factor gene homologues of *S. pneumoniae* absent in other *S. tigurinus*(–like) strains or other streptococci belonging to the Mitis group (or M-clade [20]). These virulence factors include: *cbpG*, coding for a pneumococcal cholin binding serine protease with adhesive properties; *hylA* and *hysA* homologues, coding for hyaluronidases; the pneumolysin-gene *ply*, coding for a pore-forming toxin; and the autolysin-gene *lytA*, coding for a peptidoglycan hydrolase. On the other hand, all typical (*sensu stricto*) *S. oralis* strains differ from *S. tigurinus*(–like) not only in the presence of *gtfR* (here identified as a *gtfD*–*gtfG* homologue), but also in *zmpC*, encoding a zinc-metalloproteinase involved in neutrophil extravasation, inflammation, and tissue remodeling, and in possessing *iga* encoding an immunoglobulin A1 protease, both typically found in *S. pneumoniae*. Additionally, ATCC 49296-like *S. oralis* strains lack – in comparison to all other Mitis group strains except and interestingly enough *S. tigurinus* AZ_3a – a whole range of genes responsible for rhamnose synthesis, such as *rmlA-C* and *rfbA-D*, as well as homologues responsible for the formation of streptococcal capsules, such as *wchA* or *cpsE*. ATCC 6249, differing from *S. tigurinus* and *S. oralis sensu stricto* both in *in silico* DNA–DNA hybridization (Table 2) and concatemeric tree position, possesses the genes for capsule formation but lacks rhamnose synthesis genes, a combination also found in the *S. infantis* type strain.

By analyzing the pattern of virulence associated genes *rib*-like, *int*, *cshA*-like, *pitA*-like, and *hylA*-like in the strains, the following was observed (Table 3): The *rib*-like and the integrase encoding gene were only present in the *S. tigurinus* type strain AZ_3a and in the *S. tigurinus* candidate strain SN 62386 of the collection. The *cshA-*like gene was present in all four *S. tigurinus* reference strains, as well as in four *S. oralis* and in two *S. oralis-S. tigurinus* intersectional strains, but not in any of the *S. tigurinus*–like isolates. The *pitA*-like and *hyl*A-like genes were only present in the *S. tigurinus* type strain AZ_3a and in SN 50746 of the collection.

Finally, the *gtfR*-gene distribution among *S. oralis/tigurinus/infantis* was investigated (Table 3). To confirm that *gft*R-positive strains actually do produce dextran by glycosyltransferase activity, a Mitis Salivarius Agar or Columbia Blood Agar medium was enriched with 5% sucrose. As representatively shown in Supplementary Figure S5, all of the *gtfR*-negative strains showed flat, dry, and not very entrenching colonies, whereas *gtfR*-positive strains showed much higher, rounded, water-absorbing and entrenching colonies due to strong dextran production. The following principles were found: (a) *S. tigurinus*(–like) strains did not possess the *gtfR*-gene and did not produce dextran, except SN 37569 (see Figure 1), interestingly the only *S. tigurinus*-like strain with a second 16S rRNA-operon variant holding the CTT-motif; (b) all *S. oralis sensu stricto* strains (both ATCC 35037-like and ATCC 49296-like) hold the *gtfR* gene producing dextran; (c) *S. infantis* (together with the *S. peroris* strain) did not have a *gtfR* homologue, as it is missing in ATCC 6249-like strains. The latter strain was first deposed as ‘*S. viridans*’, later reclassified as ‘*S. mitis*’, and recently reclassified as ‘*S. oralis*’, but this may not be correct either. Instead, ATCC 6249-like strains seem to form a distinct subclade, which should be referred to as *S. oralis* genomo-subspecies 1 until further description. Finally, (d) even after intensively trying to categorize the strains by several methods, some strains (SN 16495, SN 50746, and SN 63707) showed a hybrid profile.

### In situ detection of S. tigurinus variants

Testing oral samples of healthy young adults for the abundance of *S. tigurinus* (two different operon variants [AGA and CTT] were tested separately) in relation to all bacteria and all Mitis group streptococci revealed the following results: *S. tigurinus* was found in 23/35 (66%) saliva samples, with a proportion between 0.01 and 12.5% of all bacteria and between 0.05 and >90% (the latter observed in three cases) of Mitis group streptococci. The CTT-variant was only found in two samples and with low abundance. In the buccal swab samples, *S. tigurinus* was found and confirmed by amplicon sequencing only once, suggesting that this species (associated with invasiveness) is not particularly enriched on mucosal membranes. However, the buccal samples were, probably because of the self-sampling procedure chosen, not very cell rich, reducing the overall sensitivity of this investigation.

The PCR-based analysis of *S. tigurinus*–associated virulence genes in the same oral samples revealed that the *rib-*, *pitA*-, and *hyl*A-like genes, presumably representing highly virulent *S. tigurinus* strains, were absent. However, the virulence genes encoding the fibronectin-binding domain CshA and the integrase of a transposable element were found in 69 and 54%, respectively, of saliva samples, but their presence was independent of the *S. tigurinus* abundance, indicating that these factors are not species specific. Furthermore, *gtfR* – specific for *S. oralis sensu stricto* as outlined above – was found in all saliva samples.

## Discussion

Based on a Tigurinus-16S signature sequence, *S. tigurinus*–like strains were frequently found in the collection of endocarditis isolates and in databases worldwide. It became obvious from the 16S rRNA gene–based phylogenetic tree that the correct placement of strains within the Mitis group is at least partially hampered because of different 16S operon variations and numbers. According to a BLAST search and the rrn Data Base [39], the number of 16S operons within the Mitis group strains seems to vary from one (*S. oralis* ATCC 35037, *S. pseudopneumoniae* strains) to five (as in SK255). However, BLAST results may be incomplete if fewer than four operons were detected or even wrong if more than four operons were detected (possibly because of duplication during assembly process), as almost all of the genomes are not closed. In fact, all closed *Streptococcus* genomes have four rRNA operons. However, there are reports of different variants of the operons generated by recombination even between species [6].

In particular, among different operons, at least two variations of the V1 region of *S. tigurinus* and a few other Mitis group species were observed (starting with an AGA- or CTT-motif, respectively; Supplementary Figure S1). Interestingly, both variations lead to the same stem-loop structure with similar dG (–12.9 kJ·mol^−1^ in AGA vs. –16.2 kJ·mol^−1^ in the CTT version applying Mfold [40]). A simple BLAST search revealed that this V1 stem structure is hypervariable, for instance several motifs can be found among *E. coli* strains. However, as the resolution of the 16S rRNA gene is rather low for Mitis group species and as the same V1 variant occurs species independently, this may lead to phylogenetical deception.

With a few exceptions discussed below, *S. tigurinus*–like strains were only found among ‘*S. oralis*’ strains, which do not hold the glycosyltransferase-gene *gtfR*. Therefore, it is assumed that all (or at least most) of the *gtfR*-negative *S. oralis* might be *S. tigurinus*. This assumption is further supported by the fact that *S. oralis* is the only non-hemolytic *Streptococcus* species possessing *gtf*-positive and -negative strains [22]. In the study cited (from 2005) – testing 148 strains (24 taxa) of non-hemolytic streptococci – it is demonstrated that *gtf* sequences were either present in all strains of a given taxon or completely absent. As the only exception, about half (51%) of *S. oralis* possessed a *gtf* gene (*gtfR* variant). It is postulated that the other half of these *S. oralis* strains might have been (at least partially) *S. tigurinus*. Exceptions of this assumption (*gtfR* negative = *S. tigurinus*, *gtfR* positive = *S. oralis*), however, do exist in a few strains interestingly co-arising with the CTT-motif (e.g. strain SN 37569; Table 3). By further categorizing the strains, applying concatemer (16S/*gdh*/*groEL*/*sodA*) tree analysis and DNA–DNA hybridization, linker strains with a *S. oralis*–*S. tigurinus* hybrid character became recognizable (SN 16495, SN 50746, and SN 63707; Table 3), with two of them holding the CTT-motif. Such hybrids have been described before within the Mitis group, for example formed between *S. oralis* and *S. mitis* (strain SK597 [6]). Thus, a clear separation between *S. oralis* and *S. tigurinus*, preferred by whatever reason such as ‘different risks for endocarditis’, will never be sharp. Another group of strains with a comparable distance to *S. oralis* (and *S. mitis*) is for instance formed by ATCC 6249 and five endocarditis isolates of the collection according to the tree of concatenated sequences (Figure 2), the probability matrix (Table 2), as well as the MDS plot (Figure 3). This could be evidence for another ‘(sub-)species’. If so, it explains the difficulties and the inconsistency in nomenclature of this strain in the literature and databases. On the other hand, among *S. tigurinus*(–like) strains, strain 1366 and its small-colony forming derivatives 2425 and 2426 exhibited some distance not only to the *S. tigurinus* type strain AZ_3a, but also to all other clusters of the Mitis group. This was found in several other studies before including a recent study from France, where *shetA* encoding for exfoliative toxin was used as a phylogenetic marker [16]. However, recent analysis of 195 Mitis group core genome sequences showed that strain 1366 and its progenies, even with some distance to AZ_3a, clearly belong to the *S. tigurinus* cluster [7]. This study, a re-evaluation of the taxonomy of the Mitis group of the genus *Streptococcus* based on core genomes and MLSA, was contemporaneously and independently (from the present study) conducted, and came to the following analogue conclusions. First is the need for reclassification of *S. tigurinus* as *S. oralis* subsp. *tigurinus* comb. nov., with the addition that most strains do not produce extracellular polysaccharide (thus are *gtfR* and GTFR negative). In addition, they found that most of these strains also do not produce IgA1 protease. Second, strains related to ATCC 6249 form another distinct cluster within the *S. oralis* clade (*S. oralis* genomo-subspecies 1). Third, a proportion of publicly available Mitis group genomes in GenBank are incorrectly identified, which is worrying, and a critical curation is needed. Strains SK255, SK394, SK313, and SK1074 are members of the *S. tigurinus* but not *S. oralis* cluster. It should also be mentioned that according to the same study, *S. oligofermentans* strain AS 1.3089^T^, used as outgroup in the present study, was reclassified as a later synonym of *Streptococcus cristatus*.

The impossibility (or frustration) of a sharp species definition becomes even more evident when virulence genes are included into the stratification. Genes identified to be associated with high virulence in *S. tigurinus* AZ_3a [31] are not found in every *S. tigurinus* strain. To be exact and according to the *in silico* and *in vitro* analysis, *rib, pitA*, and *hylA* homologues are rare, as are *lytA* and *ply* (*in silico* analysis only, applying thresholds of sequence identity and completeness > 50%) among *S. tigurinus* strains. Finding a strain matching the *S. tigurinus* type strain AZ_3a virulence is unlikely. However, as *S. pneumoniae* is the pathogenic variant of *S. mitis*, AZ_3a-like strains may be a more virulent form within the *S. tigurinus*–*S. oralis* supercluster, and parallels in evolution should exist. Interestingly, only AZ_3a contains a collection of close homologues of *S. pneumoniae* virulence genes, in particular those coding for pneumolysin Ply together with autolysin LytA, cholin-binding protein CbpG, and hyaluronidases HylA/HysA (the latter three, however, are also found in a few other strains). This could be an explanation for its exceptional invasiveness. From a recent study [41], it is known that pneumococcal *lytA* and *ply* genes are located on a pathogenic island with a diversity of types evolved in eight steps. Their corresponding products might function together forming a (patho)physiological protein network. In addition, previous experiments have shown that the combination of *ply* and *lytA* is supporting optimal biofilm formation *in vitro*. Homologues of *ply* and *lytA* were described in *S. pseudopneumoniae*, *S. mitis*, *S. oralis*, *S. infantis*, and *S. dentisani* [41], the latter with less pathogenic [7] and possibly more probiotic potential, as it was isolated from caries-free subjects [42] and might have a caries-protective activity (López-lópez et al. [43]). This shows that in principal, *ply-lytA*-like genes are frequent, but (patho)physiologically relevant variants might be rare. The increased *ply-lytA* sequence identity and completeness (*lytA*: 99% coverage, 78% positive matches; *ply*: 99% coverage, 71% positive matches) found in *S. tigurinus* AZ_3a might be indicative for its (patho)physiological relevance. Possibly, AZ_3a evolved from a horizontal gene transfer event between a *S. tigurinus*–like strain and *S. pneumoniae*.

*S. tigurinus* strains were graduated in low- and high-virulence strains before according to *in vitro* [19,31] as well as *in vivo* [9] data. There is ample evidence that the virulence of Mitis group strains in general is very variable. For instance, in a neutropenic mouse model, the lethal infective dose (LD_50_) of bacterial cells of different *S. mitis* strains varied between 1.9 × 10^4^ CFU and 1.6 × 10^6^ CFU [44]. Likewise, the infective dose (IF_90_) for the *S. tigurinus* AZ_3a (and AZ_14) tested in rats was 10^4^ CFU, but that of *S. tigurinus* strain AZ_8 was higher, emphasizing intraspecies virulence variability. Very recently, strain AZ_8 was confirmed as low virulent by pathogenomic profiling [31].

The prevalence and abundance of *S. tigurinus* was further investigated in the oral specimens of 35 volunteers. The prevalence found was 66%. The prevalence had been determined before but with inconsistent results. Zbinden et al. [10], screening saliva specimens of 31 volunteers by selective growth, MALDI-TOF MS, and subsequent 16S-sequencing, did not identify *S. tigurinus* among 608 isolates. In contrast, the same group found the species in roughly 50% of both periodontitis cases and controls by applying TaqMan PCR in a later study [13]. From oral samples, culture-based methods are limited in accurate selection (because of the absence of a typical colony morphology and the dominance of *S. mitis*), but also identification of *S. tigurinus* (including MALDI-TOF MS as tested by Isaksson et al. [45]). Thus, an underestimation of *S. tigurinus* in the oral cavity is likely by choosing a culture-based approach. Concerning *S. tigurinus* abundance, it was found that it can be very high in a few individuals, reaching >90% of Mitis group streptococci. The present study is also the first to screen oral samples directly for *S. tigurinus* genes presumably associated with high virulence. From the results – even if not testing all relevant genes – it can be excluded that strains with a type-strain-like virulence pattern are frequent.

In conclusion and visualized in the MDS plots of genomic as well as concatemer data (Figure 3), ample evidence was found that some species within the Mitis group are more confluent than distinct, especially comparing *S. oralis* and *S. tigurinus* clusters forming ‘two halves of one coffee bean’. Moreover, hybrid strains can be expected that do not fit into man-made pigeonholes. However, though closely related, *S. oralis* and *S. tigurinus* can be genetically and phenotypically separated by the presence or absence of *gtfR* and dextran, as well as IgA protease production. Taken together, the present results challenge the current species concept within the Mitis group, and *S. tigurinus* is no exception here. Another consequence is that the species name given to a strain has almost no predictive value for its virulence gene arsenal and especially not for the actual expression of these genes. It is recommended that those Mitis group strains causing a fatal clinical outcome should be sent to a reference laboratory for pathogenomic profiling. Furthermore, the present data underline that Mitis group streptococci harbor an exceptional ‘talent’ for recombination, diversification, and – ultimately – evolution.

## Supplementary Material

Supplementary Material

## Supplementary Material

The supplemental material for this article can be accessed here.

## References

[CIT0001] Oren A, Garrity GM. (2014). Then and now: a systematic review of the systematics of prokaryotes in the last 80 years. Antonie Van Leeuwenhoek.

[CIT0002] Stackebrand E, Goebel BM (1994). Taxonomic note: a place for DNA-DNA reassociation and 16S rRNA sequence analysis in the present species definition in bacteriology. Int J Syst Evol Microbiol.

[CIT0003] Stackebrand E, Ebers J (2006). Taxonomic parameters revisited: tarnished gold standards. Microbiol Today.

[CIT0004] Conrads G, Claros MC, Citron DM (2002). 16S-23S rDNA internal transcribed spacer sequences for analysis of the phylogenetic relationships among species of the genus *Fusobacterium*. Int J Syst Evol Microbiol.

[CIT0005] Bishop CJ, Aanensen DM, Jordan GE (2009). Assigning strains to bacterial species via the internet. BMC Biol.

[CIT0006] Kilian M, Poulsen K, Blomqvist T (2008). Evolution of *Streptococcus pneumoniae* and its close commensal relatives. PLoS One.

[CIT0007] Jensen A, Scholz CF, Kilian M (2016). Re-evaluation of the taxonomy of the Mitis group of the genus *Streptococcus* based on whole genome phylogenetic analyses, and proposed reclassification of *Streptococcus dentisani* as *Streptococcus oralis* subsp. *dentisani* comb. nov., *Streptococcus tigurinus* as *Streptococcus oralis* subsp. *tigurinus* comb. nov., and *Streptococcus oligofermentans* as a later synonym of *Streptococcus cristatus*. Int J Syst Evol Microbiol.

[CIT0008] Kanamori H, Kakuta R, Yano H (2015). A case of culture-negative endocarditis due to *Streptococcus tigurinus*. J Infect Chemother.

[CIT0009] Veloso TR, Zbinden A, Andreoni F (2013). *Streptococcus tigurinus* is highly virulent in a rat model of experimental endocarditis. Int J Med Microbiol.

[CIT0010] Zbinden A, Mueller NJ, Tarr PE (2012). *Streptococcus tigurinus* sp. nov., isolated from blood of patients with endocarditis, meningitis and spondylodiscitis. Int J Syst Evol Microbiol.

[CIT0011] Miyazato A, Ohkusu K, Tachi Y (2014). Two cases of infective endocarditis caused by *Streptococcus tigurinus*. Kansenshogaku Zasshi.

[CIT0012] Dhotre SV, Mehetre GT, Dharne MS (2014). Isolation of *Streptococcus tigurinus* - a novel member of *Streptococcus mitis* group from a case of periodontitis. FEMS Microbiol Lett.

[CIT0013] Zbinden A, Aras F, Zbinden R (2014). Frequent detection of *Streptococcus tigurinus* in the human oral microbial flora by a specific 16S rRNA gene real-time TaqMan PCR. BMC Microbiol.

[CIT0014] Hirai J, Sakanashi D, Hagihara M (2016). Bacteremia due to *Streptococcus tigurinus*: a case report and literature review. J Infect Chemother.

[CIT0015] Michelena A, Bonavila C, Zubeltzu B (2015). Endocarditis due to *Streptococcus tigurinus*: presentation of a case and a review of the literature. Enferm Infecc Microbiol Clin.

[CIT0016] Peuchant O, Wirth G, Tixier R (2016). Infective endocarditis caused by *Streptococcus tigurinus*-like organisms. New Microbes New Infect.

[CIT0017] Bourassa L, Clarridge JE (2015). Clinical significance and characterization of *Streptococcus tigurinus* isolates in an adult population. J Clin Microbiol.

[CIT0018] Gizard Y, Zbinden A, Schrenzel J (2013). Whole-Genome sequences of *Streptococcus tigurinus* Type Strain AZ_3a and S. tigurinus 1366, a strain causing prosthetic joint infection. Genome Announc.

[CIT0019] Zbinden A, Quiblier C, Hernandez D (2014). Characterization of *Streptococcus tigurinus* small-colony variants causing prosthetic joint infection by comparative whole-genome analyses. J Clin Microbiol.

[CIT0020] Zheng W, Tan TK, Paterson IC (2016). StreptoBase: an oral *Streptococcus mitis* group genomic resource and analysis platform. PLoS One.

[CIT0021] Zbinden A, Bostanci N, Belibasakis GN (2015). The novel species *Streptococcus tigurinus* and its association with oral infection. Virulence.

[CIT0022] Hoshino T, Fujiwara T, Kilian M (2005). Use of phylogenetic and phenotypic analyses to identify nonhemolytic streptococci isolated from bacteremic patients. J Clin Microbiol.

[CIT0023] Teng LJ, Hsueh PR, Tsai JC (2002). groESL sequence determination, phylogenetic analysis, and species differentiation for viridans group streptococci. J Clin Microbiol.

[CIT0024] Meier-Kolthoff JP, Klenk HP, Goker M (2014). Taxonomic use of DNA G+C content and DNA-DNA hybridization in the genomic age. Int J Syst Evol Microbiol.

[CIT0025] Demsar J, Curk T, Erjavec A (2013). Orange: data mining toolbox in python. J Machine Learn Res.

[CIT0026] Stalhammar-Carlemalm M, Stenberg L, Lindahl G (1993). Protein rib: a novel group B streptococcal cell surface protein that confers protective immunity and is expressed by most strains causing invasive infections. J Exp Med.

[CIT0027] Wastfelt M, Stalhammar-Carlemalm M, Delisse AM (1997). The Rib and alpha proteins define a family of group B streptococcal surface proteins that confer protective immunity. Adv Exp Med Biol.

[CIT0028] Jenkinson HF, McNab R, Holmes AR (1997). Function and immunogenicity of cell-wall-anchored polypeptide CshA in oral streptococci. Adv Exp Med Biol.

[CIT0029] McNab R, Jenkinson HF, Loach DM (1994). Cell-surface-associated polypeptides CshA and CshB of high molecular mass are colonization determinants in the oral bacterium *Streptococcus gordonii*. Mol Microbiol.

[CIT0030] Bagnoli F, Moschioni M, Donati C (2008). A second pilus type in *Streptococcus pneumoniae* is prevalent in emerging serotypes and mediates adhesion to host cells. J Bacteriol.

[CIT0031] Diene SM, Francois P, Zbinden A (2016). Comparative genomics analysis of *Streptococcus tigurinus* strains identifies genetic elements specifically and uniquely present in highly virulent strains. PLoS One.

[CIT0032] Liu WT, Marsh TL, Cheng H (1997). Characterization of microbial diversity by determining terminal restriction fragment length polymorphisms of genes encoding 16S rRNA. Appl Environ Microbiol.

[CIT0033] Molbak L, Klitgaard K, Jensen TK (2006). Identification of a novel, invasive, not-yet-cultivated *Treponema* sp. in the large intestine of pigs by PCR amplification of the 16S rRNA gene. J Clin Microbiol.

[CIT0034] Chalmers NI, Palmer RJ, Cisar JO (2008). Characterization of a *Streptococcus* sp.-*Veillonella* sp. community micromanipulated from dental plaque. J Bacteriol.

[CIT0035] Glazunova OO, Raoult D, Roux V (2009). Partial sequence comparison of the rpoB, sodA, groEL and gyrB genes within the genus *Streptococcus*. Int J Syst Evol Microbiol.

[CIT0036] Peyyala R, Kirakodu SS, Ebersole JL (2011). Novel model for multispecies biofilms that uses rigid gas-permeable lenses. Appl Environ Microbiol.

[CIT0037] Nadkarni MA, Martin FE, Jacques NA (2002). Determination of bacterial load by real-time PCR using a broad-range (universal) probe and primers set. Microbiology.

[CIT0038] Tamura K, Stecher G, Peterson D (2013). MEGA6: molecular evolutionary genetics analysis version 6.0. Mol Biol Evol.

[CIT0039] Stoddard SF, Smith BJ, Hein R (2015). rrnDB: improved tools for interpreting rRNA gene abundance in bacteria and archaea and a new foundation for future development. Nucleic Acids Res.

[CIT0040] Zuker M (2003). Mfold web server for nucleic acid folding and hybridization prediction. Nucleic Acids Res.

[CIT0041] Morales M, Martin-Galiano AJ, Domenech M (2015). Insights into the evolutionary relationships of LytA autolysin and Ply pneumolysin-like genes in *Streptococcus pneumoniae* and related streptococci. Genome Biol Evol.

[CIT0042] Camelo-Castillo A, Benitez-Paez A, Belda-Ferre P (2014). *Streptococcus dentisani* sp. nov., a novel member of the mitis group. Int J Syst Evol Microbiol.

[CIT0043] López-lópez A, Camelo-castillo AJ, Ferrer MD, et al (2017).

[CIT0044] Shelburne SA, Sahasrabhojane P, Saldana M (2014). *Streptococcus mitis* strains causing severe clinical disease in cancer patients. Emerg Infect Dis.

[CIT0045] Isaksson J, Rasmussen M, Nilson B (2015). Comparison of species identification of endocarditis associated viridans streptococci using rnpB genotyping and 2 MALDI-TOF systems. Diagn Microbiol Infect Dis.

