# Supplementary material for: Streptococcus tigurinus is frequent among gtfR-negative Streptococcus oralis isolates and in the human oral cavity, but highly virulent strains are uncommon
Source: J Oral Microbiol. 2017 Apr 20;9(1):1307079. doi: 10.1080/20002297.2017.1307079 (PMC5405715; doi:10.1080/20002297.2017.1307079)
Supplement: Supplementary Material [file zjom_a_1307079_sm6785.docx]

**Supplementary Material**

*Fig. S1 (Supplementary).* Comparison between a 16S rRNA-V1-region of *S. tigurinus* type strain AZ_3a (left, dominating variant, dG= -12.9 kJ·mol^−1^) and of *S. tigurinus* 1366 (right, sporadically found in *S. tigurinus* and a few other species as framed in **Fig. 1**, dG= -16.2 kJ·mol^−1^). Folding and dG calculation was done by mfold (44).

*Fig. S2 (Supplementary).* Phylogenetic tree based on the *gdh*-gene of Mitis group streptococci. Program MEGA6, Maximum likelihood algorithm subsequently to ClustalW-alignment, standard preferences, 100 bootstraps (33); ● SN-strains of our collection; in strain SN 57625 the *gdh*-gene could not be amplified and sequenced. *Streptococcus oligofermentans* strain AS 1.3089^T^ was used as outgroup.

*Fig. S3 (Supplementary).* Phylogenetic tree based on the *groEL*-gene of Mitis group streptococci. Program MEGA6, Maximum likelihood algorithm subsequently to ClustalW-alignment, standard preferences, 100 bootstraps (33); ● SN-strains of our collection. *Streptococcus oligofermentans* strain AS 1.3089^T^ was used as outgroup.

*Fig. S4 (Supplementary).* Phylogenetic tree based on the *sodA* -gene of Mitis group streptococci. Program MEGA6, Maximum likelihood algorithm subsequently to ClustalW-alignment, standard preferences, 100 bootstraps (33); ● SN-strains of our collection. *Streptococcus oligofermentans* strain AS 1.3089^T^ was used as outgroup.

*Fig. S5 (Supplementary).* Comparison between dextran producer (left, *gtfR* and GtfR-positive, *S. oralis* *sensu stricto*) and non-producer (right, *gtfR* and GtfR-negative, *S. tigurinus*) strains after growth on Mitis-Salivarius agar with additional 5% sucrose for 48 hours anaerobically. Magnification 10 x.

Figure S1 (supplementary): comparison of V1 regions


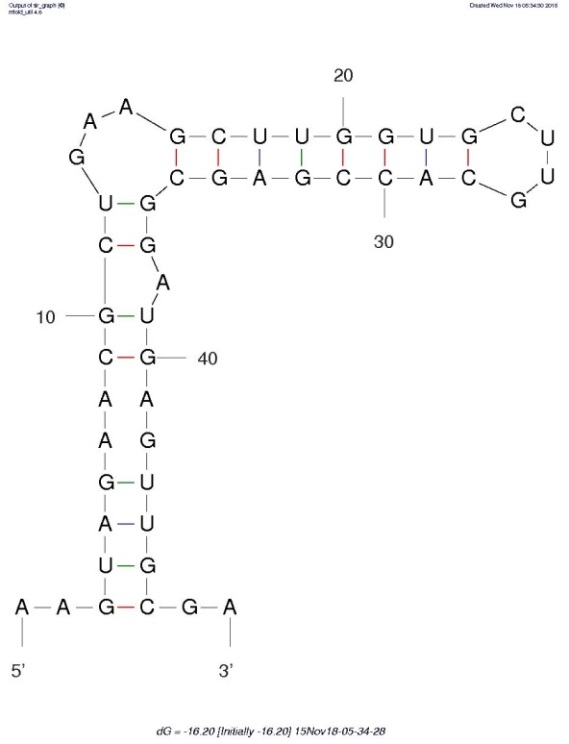

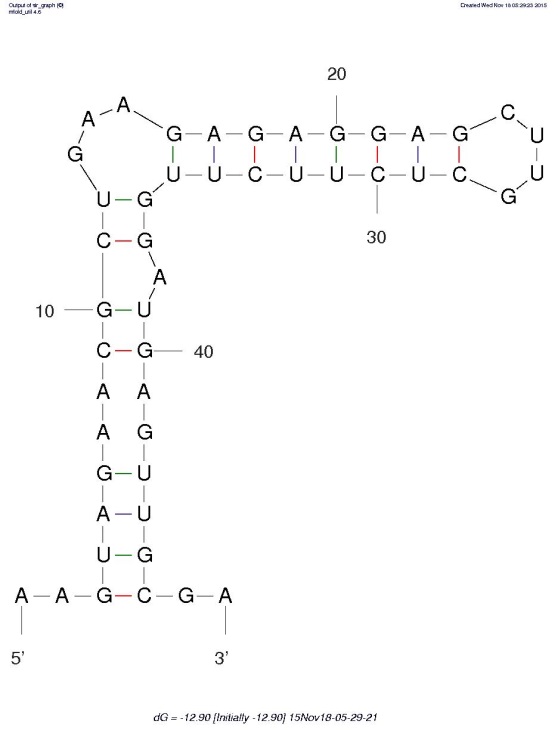

 Figure S2 (supplementary): *gdh*-tree

Figure S3 (supplementary): *groEL*-tree

Figure S4 (supplementary): *sodA*-tree

Figure S5 (supplementary): dextran production
